# The ^1^H NMR Profile of Healthy Dog Cerebrospinal Fluid

**DOI:** 10.1371/journal.pone.0081192

**Published:** 2013-12-23

**Authors:** Mihai Musteata, Alina Nicolescu, Gheorghe Solcan, Calin Deleanu

**Affiliations:** 1 Clinics Department, Faculty of Veterinary Medicine, University of Agricultural Science and Veterinary Medicine Iasi, Romania; 2 Group of Biospectroscopy, Institute of Macromolecular Chemistry, Roumanian Academy, Iasi, Romania; 3 Group of Biospectroscopy, Centre of Organic Chemistry, Roumanian Academy, Bucharest, Romania; Biological Research Centre of the Hungarian Academy of Sciences, Hungary

## Abstract

The availability of data for reference values in cerebrospinal fluid for healthy humans is limited due to obvious practical and ethical issues. The variability of reported values for metabolites in human cerebrospinal fluid is quite large. Dogs present great similarities with humans, including in cases of central nervous system pathologies. The paper presents the first study on healthy dog cerebrospinal fluid metabolomic profile using ^1^H NMR spectroscopy. A number of 13 metabolites have been identified and quantified from cerebrospinal fluid collected from a group of 10 mix breed healthy dogs. The biological variability as resulting from the relative standard deviation of the physiological concentrations of the identified metabolites had a mean of 18.20% (range between 9.3% and 44.8%). The reported concentrations for metabolites may be used as normal reference values. The homogeneity of the obtained results and the low biologic variability show that the ^1^H NMR analysis of the dog’s cerebrospinal fluid is reliable in designing and interpreting clinical and therapeutic trials in dogs with central nervous system pathologies.

## Introduction

Metabolomics, one of the young “omics” sciences studies small molecules (metabolites) simultaneously from various natural occurring samples (plants or animals, fluids or tissues, leaving or extracts, etc). The term metabolomics was first introduced by Oliver [1], and later refined by Nicholson [2,3] to distinguish between a static snap-shot of several metabolites (metabolomics) *versus* a time related dynamic study of the metabolite concentrations (metabonomics). 

The simultaneous determination of concentrations for several metabolites investigated in metabolomics and metabonomics relies on complex analytical techniques (NMR, HPLC, MS, GS, etc), including hyphenated ones (GC-MS, LC-MS, LC-NMR-MS, etc). There are many reviews describing applications in various fields, definitions and history of the field [4,5].

^1^H NMR spectroscopy offers several advantages as compared with classical methods. Thus, the ^1^H NMR spectrum represents the global profile of the analyzed sample, making possible the identification and quantification of more compounds, without the need for individual isolation. A small volume of sample is required for the NMR analysis, from 1 mL down to few microliters with the existing state-of-the-art accessories (e.g. cryoprobes). There is very simple or no sample preparation required for NMR analysis of biological fluids, thus only addition of a small volume of deuterated water (usually 10% volume), with or without centrifugation being needed. If desired, the pH can be adjusted with buffer solutions or acids / bases solutions. For longer time storage, a preservative (e.g. sodium azide) was added to the sample, to reduce chemical deterioration until NMR analysis. 

The obtained spectra contain a large amount of information that can be stored for further analysis. A part of the information obtained through ^1^H NMR spectroscopy analysis can be used for transforming it in individual concentrations of some metabolites [6], whereas the whole data set may be used for group classifications [7,8]. 

Cerebrospinal fluid is a product of plasma filtration and membrane secretion, with a different composition from the blood plasma [9-11], being dependent on the cerebral metabolic rate and being involved in the transport of nutrients and waste from the neuronal metabolic processes.

Valuable information concerning the CSF metabolome have been obtained as a result of the analysis through different complex techniques (HPLC, NMR, MS, GS, etc.). These techniques made possible the determination of some compounds which are not usually quantified through classic methods of analysis [6,12-14]. Mandal et al. [15] investigating the human CSF metabolome, through multiplatform analysis have assessed that increasing the number of identified metabolites is only possible by combining a number of techniques (^1^H NMR, MS, GS, HPLC, etc.) as long as no essential improvements can be brought to each of the already existing techniques. Several groups identified markers for hemorrhagic accidents [4,16-19], degenerative diseases [20,21], psychiatric disorders [22], and analyzed differences in known metabolites concentration in some neurological pathologies [23,24]. Also, it was evaluated the impact of the non-neurological diseases on CNS [25-28]. The study of CSF biomarkers is also very important in the evaluation of the pharmacokinetics of some new drugs [29-32]. 

The identification and labelling of the molecular compounds of CSF as biomarkers is limited by some objective aspects [33] especially when the samples are collected form healthy humans. The study of CSF from animals raises some problems related to the validation of the identified compounds with the ones already investigated in humans, but the requests concerning the studied individuals’ homogeneity and implicitly their analysis results are fulfilled. 

Among all animal species, the dog presents a great number of similarities with humans, many of the pathological conditions of the human CNS being also met in dogs and both seem to have common pathogenesis. Experimental [34-39] and clinical [40,41] research undertaken on dogs CSF led to the identification of biomarkers present in the human CSF. 

Although in experimental studies various metabolites from dogs’ CSF were identified and analyzed, to our knowledge no study has simultaneously quantified several metabolites from CSF of healthy dogs. The present study aims to present the quantitative and qualitative assessment of the dogs’ CSF compounds, using ^1^H NMR spectroscopy, and to consider the presence of those species-specific particularities. The paper also aims to analyse, in a comparative manner, the rate of biological variability of the identified metabolites and the data existing in previously reported studies. In order to achieve these objectives, NMR spectroscopy was considered as the most versatile and suitable method for identification and quantification of individual metabolites. Moreover, once more date will become available from additional and larger cohorts of healthy dogs, the present NMR data may be integrated with future ones and used for untargeted metabolomic approaches based on the entire NMR spectral envelope. 

## Materials and Methods

### Subjects and sample collection

The study was approved by the Council of Ethics of the University of Agricultural Sciences and Veterinary Medicine from Iasi Romania (839/27.09.2011) and the owners of the dogs gave the permission for their use in the study. The samples of cerebrospinal fluid were harvested from 10 healthy, adult mixed breed dogs of both sexes (2-5 years of age and 15-20 Kg), without neurologic disease history. The CSF sampling was taken by puncture at the atlantooccipital space, under general anaesthesia with medetomidine (Domitor, Pfizer) 0.03 mg/bw and Ketamine (Ketamine, Kepra) 0.1 mg/bw inj. IV. All samples were taken during the same time interval: 10.00-12.00 a.m. dogs being fed normally until previous evening and fasten during the night. The samples were distributed into two vials, one for the NMR analysis and one for the routine cytological and protein analysis. The cytological exam was undertaken immediately after the sampling, in order to prevent cellular degradation. Aliquots of 2 mL were stored at –80 °C until the NMR analysis was performed.

### Sample preparation

Before NMR analysis the CSF samples were allowed to reach room temperature (typically for 30 minutes) and were centrifuged at 7,000 rotations per minute for 10 minutes. The samples were prepared by transferring a 540 μL aliquot of CSF to a 1.5 mL Eppendorf tube followed by the addition of 60 μL phosphate buffer solution (pH 7.5) in deuterated water (D_2_O). The buffer solution also contained 5 mM sodium 3-(trimethylsilyl)-[2,2,3,3-d4]-1-propionate (TSP, used as an internal standard of known concentration) and sodium azide (used as preservative). The final sample pH was 7.0. The entire volume of sample (600 μL) were transferred into a 5 mm NMR tube and subjected to the analysis. 

### NMR measurements

^1^H NMR spectra were acquired at 26.5°C, on a Bruker Avance III 400 MHz spectrometer, operating at 400.13 MHz, using a 5 mm inverse detection probe equipped with gradients on the *z*-axis. The samples were run in 5 mm Wilmad 507 NMR tubes. The spectra were recorded with the NOESY presaturation pulse sequence using 32 scans, 30 s relaxation delay, 4 s acquisition time, 8223 Hz spectral window, collecting 64 K data points, with a digital resolution of 0.12 Hz. An exponential line broadening factor of 0.3 Hz was used in post-acquisition FID processing. The chemical shifts are reported as δ values (ppm) referred to TSP (0.0 ppm) as internal standard. The spectra were acquired and processed with the Bruker TopSpin 2.1pl6 software. The NMR protocol allows full relaxation of signals over a 37 s overall relaxation delay, as previously employed by us for other biological samples [42-45]. Thus, with 0.3 Hz line broadening and 37 s relaxation delay, signal intensities may be employed for quantitation purposes. For crowded NMR spectra, using signal intensities instead of integrals is less prone to errors induced by partial superposition of signals. As the amount of collected CSF was kept to minimum necessary, one NMR analysis was performed with 600 µL of sample (the regular 5 mm NMR filling volume). Employing one measurement with regular filling volume instead of two measurements with half filling volume produces superior results due to both higher sensitivity (higher signal to noise ratio) and to better shimming (better magnetic field homogeneity leading to sharper lines in the spectrum). In order to keep the operator error to a minimum, only one expert operator performed all NMR sample preparations and experiments. For the same operator the analytical error was checked and shown to be well below the biological variation. Thus, for six different NMR samples prepared from the same original batch, the analytical error for various metabolites was ranging between 1-5%, whereas the biological variation (as % RSD) was in the range of 10-45%. 

### Statistical analysis

Data were processed using Statistical Package for the Social Science (SPSS) 16.0 for Windows. The obtained values are presented as means with standard deviation. Distributions were analysed for each metabolites with Shapiro-Wilk test, and α-level of less than 0.05 was regarded as significant. The biologic variability of the identified metabolites was assessed by analysing the relative standard deviation (RSD). The total biological variability was represented by the RSD mean for each metabolite. Pearson correlations were also computed between the metabolites concentration.

## Results

**Figure 1:**
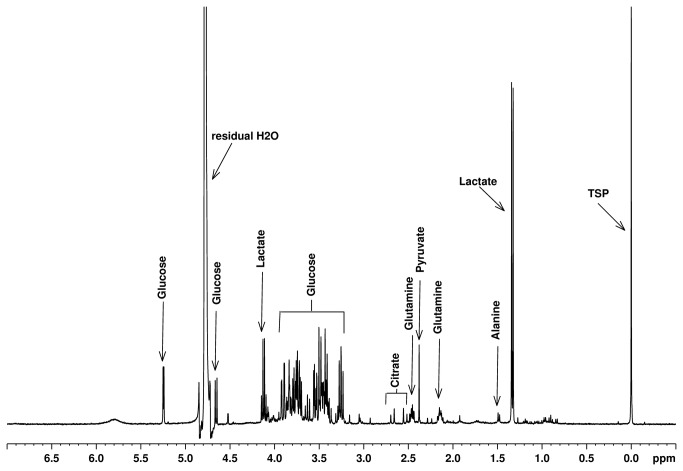
^1^H NMR spectrum of dog CSF. Some of the major metabolites are labelled.

All CSF samples were considered to be normal after cytological and protein examination. A typical ^1^H NMR spectrum of CSF from one healthy dog is presented in Figure 1. 

The typical ^1^H NMR spectrum of dog CSF is crowded, with overlapping of signals corresponding to different metabolites. Using several NMR signals we identified and quantified 13 metabolites. The obtained NMR spectra are similar with the NMR spectra of human CSF previously reported in the literature [4,46]. Thus, some of the signals were assigned according to these reports and were further verified by 2D NMR experiments (H-H COSY and J-Resolved). Eleven metabolites were present in all dogs, with valine and acetone present in only 6 and 9 dogs respectively. The metabolites concentrations were calculated using the signals intensities. The obtained values are presented in Table 1. For each metabolite one or more signals were used as indicated in Table 1.

**Table 1:** The metabolites identified in dogs CSF through ^1^H NMR compared with values previously reported in humans.

Metabolite	Signal’s chemical shifts (ppm)	Determined values Mean ± Standard Deviation (mmol/L)	Previously reported reference values in dogs (mmol/L)	Previously reported reference values in humans (HMDB) (mmol/L)
Creatinine	3.05	0.044 ± 0.004	0.0442 ^[^^35^^†^^]^	0.226 ± 0.042 ^[^^6^ 0.043 ± 0.012 ^[^^15^^]^
α-hydroxy-butyrate	0.90	0.062 ± 0.016	-	0.041 ± 0.014 ^[^^6^ 0.040 ± 0.024 ^[^^4,15^^]^
Valine	0.99; 1.05	0.027 ± 0.006	0.004 ± 0.0001^[^^57^^#^^]^	0.019 ± 0.013^[^^4,15]^ 0.02 (0.01-0.03) ^[^^14^^]^
Lactate	1.33	2.094 ± 0.374	1.90 ± 0.61 ^[34§]^	1.688 ± 0.376 ^[6]^ 1.651 ± 0.626 ^[^^4,15]^ 1.5 ^[^^58]^
Alanine	1.48	0.072 ± 0.010	0.0075 ± 0.003^[57#]^	0.046 ± 0.027 ^[4,15]^
Acetic acid	1.92	0.036 ± 0.007	-	0.058 ± 0.027 ^[4,15]^ 0.284 ± 0.126 ^[59]^ 0.100 ± 0.030 ^[60]^
Acetone	2.23	0.029 ± 0.013	-	0.020 ± 0.021 ^[4,15]^
Pyruvate	2.38	0.235 ± 0.037	0.19 ± 0.07 ^[34§]^	0.046 ± 0.016 ^[6]^ 0.053 ± 0.042 ^[4,15]^ 0.110 ^[^^58]^ 0.115 ± 0.017 ^[59]^
Glutamine	2.43-2.86	0.419 ± 0.039	0.431 - 0.500 ^[36‡]^	0.388 ± 0.079 ^[6]^ 0.432 ± 0.204 ^[4]^
Citrate	2.54; 2.68	0.124 ± 0.023	0.004 ± 0.0001 ^[57#]^	0.552 ± 0.141 ^[6]^ 0.225 ± 0.096 ^[4]^ 0.370 (0.11-0.63) ^[14]^ 0.400 ± 0.170 ^[60]^
myo-inositol	4.08	0.500 ± 0.054	-	0.164 ± 0.038 ^[6]^ 0.084 ± 0.040 ^[4]^
Ascorbic acid	4.52	0.255 ± 0.041	-	0.133 ± 0.0588 ^[61]^ 0.164 ± 0.021 ^[62]^
Glucose	5.25	3.988 ± 0.427	3.58 ± 0.47 ^[34‡]^3.679 ± 0.288 ^[35†]^	3.732 ± 0.699 ^[6]^ 2.96 ± 1.11 ^[4]^ 1.72 (1.56-1.88) ^[59]^ 5.39 ± 1.65 ^[60]^

***^†^*** - technique not-specified, ***^‡^*** - HPLC examination, ***^§^*** - lactate oxidase methodology, ***^#^*** - cromatography

The RSD values of the identified metabolites were ≤ 10% for 2 metabolites, between 10 and 20% for 7 metabolites, between 20 and 30% for 3 metabolites and greater than 30% for only one metabolite. The RSD values obtained for the identified compounds are presented in Table 2. The RSD of the identified metabolites concentration had a mean of 18.2% with a minimum of 9.3%, and a maximum of 44.8%.

**Table 2:** Biologic variability (RSD) of the identified metabolites in dog CSF.

Metabolite	Determined RSD (%)
Creatinine	10.00
α-hydroxy-buthirate	25.13
Valine	23.47
Lactate	17.87
Alanine	13.64
Acetic acid	20.33
Acetone	44.81
Pyruvate	15.79
Glutamine	9.32
Citrate	18.82
myo-inositol	10.82
Ascorbic acid	15.97
Glucose	10.69

The data analysis using the Pearson test showed a high degree of correlation between pyruvate and lactate (*r* = 0.794, *P* = 0.006), ascorbic acid and α-hydroxybutyrate (*r* = 0.779, *P* = 0.008). Significant correlations were also noticed in the case of pyruvate – alanine (*r* = 0.665, *P* = 0.036), citrate – glutamine (*r* = 0.700, *P* = 0.024), myo-inositol – ascorbic acid (*r* = 0.704, *P* = 0.023) and valine – α-hydroxybutyrate (*r* = 0.855, *P* = 0.030).

## Discussion

The ^1^H NMR spectra of CSF offer an overview of the CSF profile and thus of the cerebral metabolism. The identified compounds are involved in most of the brain metabolism processes: glutamine (glutamatergic system), myo-inositol (second messenger pathways), glucose, ascorbate, lactate, pyruvate, valine, creatinine (energy metabolism), citrate (calcium / energy metabolism), α-hydroxybutyrate and acetone (fatty acid metabolism).

The quantitative analysis of the 13 metabolites in the present study showed closely resembling values with the ones registered in humans [15,47], the differences being generally represented only by the biological variation. The level of biological variation has a great importance independently of the mean value. Thus, in humans at a high RSD, it was proved that for many of the identified metabolites it is no longer possible to discriminate the samples of normal CSF from those harvested from patients with different neurological pathologies. In such a situation, Stoop et al. [48] concluded that CSF is more influenced by the differences among individuals (person to person) than by the effect of a specific disease which may have a minor contribution. He obtained a reduced biological variation (< 20%) only for the abundant metabolites (lactate, glucose), and only for a few of those with an average abundance (citric acid, glutamine, acetone). Some authors [48] underlined that for most of the compounds with average concentrations, RSD was up to 30%. In humans, they got the most reduced biological variation of the metabolites, ranging between 15 and 70%, better than in other studies [4,15,49]. This underlines the fact that the biological variation of the metabolites in CSF is directly influenced by the characteristics of the individuals’ group from whom the samples were harvested. A homogeneous sample group (considering age, sex, health state etc) is the easiest way of lowering the biological variation of metabolites. In our case, for dogs, the variation was between 9.3% and 44.8%, with a mean of 18.2% being much smaller than the previous studies on humans, demonstrating the direct effect of sampling. Excepting for lactate and acetone, the RSD of the metabolites that have been followed by us in dogs are much lower in comparison with the ones described before in humans. Thus, 9 of the 13 studied metabolites had an RSD smaller than 20%. An RSD greater than 30% was obtained in the case of only one compound (acetone, i.e. 44.81%). Referring to acetone, there are only few reports on this metabolite in CSF and ^1^H NMR examination was the only method through which this metabolite was detected in CSF [48]. Previous reports indicated higher concentrations of acetone in CSF in patients with amyotrophic lateral sclerosis [24] and also as much as 100% variation in human

s[4]. 

The most important factors influencing the concentration of CSF metabolites (and implicitly their RSD) were supposed to be genetics, breeding, diet, environment and age [49]. The age impact on the quantitative profile of the CSF was already demonstrated in comprehensive studies of metabolomics and proteomics [48]. Concerning the age and weight, the group of dogs included in the present study was homogeneous: the age difference among individuals was of 3 years and the weight difference of maximum 5 Kg (all dogs being of medium size). Moreover, the sampling being made during the same time slot, the possible circadian influences on the CSF profile [55] was reduced. It should be noted that the 3 years age range selected by us, is described as adult age in dog species [50]. If we compare the results with those from human studies [4,6,50,51], the large RDS obtained in humans (with similar number of individuals) may be the result of a larger range of ages and the difficulty in obtaining a homogenous group. Interestingly, although samples were collected from mixed breed dogs, the RDS was low. Regarding body weight, although the range was between 15 and 20 Kg, we obtained values for some metabolites similar to those previously described in which dogs with different weight or from different breeds were studied [34,35,52]. Thus, in spite of the fact that dog is a mammalian with large size and genetic variability this pilot study on mixed breed dogs may be used as reference values for further studies. 

An important aspect that could be assessed in this study is the individual’s health status. The difficulty to obtain CSF samples from healthy humans, limited the reference values to patients with meningitis suspicion [4], unconfirmed neurological pathology [15,52,53], patients with neuropsychiatric pathology [6], or patients with non-neurological pathology [48] as well as to some patients with no pathology (but without quantitative determinations of the metabolites) [49]. These aspects have a great importance in the interpretation of results as well as in the attempt to establish the quality of biomarker for an identified compound. In our case, all dogs were healthy individuals. Considering the much larger reported RDS in humans (mainly due to scarcity of true control cases), this pilot study on control dogs should make the assessment of pathological conditions in dogs easier to spot based on data reported here. However, further studies on control dogs are necessary in order to asses if there is a larger normal variation of concentrations and if such variations may be associated with various breeds.

The correlations between the determined metabolites in dogs were similar with those reported by the studies performed on human CSF in physiologic conditions. Specific differences were observed in cases of data obtained in studies on human pathologic CSF. Levine et al. [54] comparing the CSF samples from humans with or without depression have observed a negative correlation between glutamine and hydroxybutyrate. In the samples analyzed in this study it was found a positive correlation between the α–hydroxybutyrate and glutamine, but with no statistical significance (*r* = 0.623, *P* = 0.462). In addition to that, the glutamine was significantly correlated with the lactate in human multiple sclerosis [52] as an effect of the modifications produced at the astrocitar level. In normal conditions there was no registered correlation, in the case of dogs (*r* = 0.437, *P* = 0.206).

The ratio lactate/pyruvate (L/P) is considered to be an indicator of the redox cerebral status. However, this ratio was considered without relevance in dogs in some circumstances [55]. This statement is also supported by our results in which the ratio lactate/pyruvate did not correlate with either lactate, pyruvate, or any other metabolite. This proves that the cerebral energetic status is more precisely determined when the lactate and pyruvate are analyzed individually in correlation with other biomarkers. A similar situation was found in the case of the correlation between the lactate and glucose. Levine et al. [56], on the other hand, noticed a significant negative correlation (*r* = - 0.73, *P* < 0.000001) unlike the one noticed by us where the correlation was positive with no statistical significance (*r* = 0.623, *P* = 0.055).

Sometimes, in physiological conditions, it is difficult to assign metabolites to a particular metabolism sequence because of the existence of multiple interferences among the metabolic ways and of the involvement of a large number of regulating enzymes. The analysis of CSF through ^1^H NMR contributed to the simultaneous identification of a great number of compounds, thus helping in the integrated analysis of the physiological relations among various systems and metabolic ways of the CSF. Although the study was made on a limited number of dogs, the ^1^H NMR derived concentrations allowed correlations between various metabolites. This validates the results by highlighting interconnections of metabolic pathways previously described in humans as well as those specific in dogs. 

To our knowledge there is no previous study undertaken on dogs CSF considering simultaneously 13 metabolites for quantification and correlations among them. The obtained data offered the possibility for higher fidelity analysis of the cerebral metabolic profile and for the assessment of CSF homeostasis in healthy dogs. The reduced degree of metabolites variability and the great number of simultaneously identified metabolites demonstrate the fact that the ^1^H NMR analysis has an important potential for further research of the CSF of canine patients with neurological, or even other types of pathologies. 

Data reported in this study may be used as reference values for further studies of CSF metabolomics in dogs and contribute to the understanding of dog metabolism. Moreover, due to the closely resembling interspecies metabolites concentrations, shorter lifespan and great similarities with the pathogenesis of human CNS disease, canine species is a valuable alternative in clinical research of the human CNS. Further studies with other control dogs and possibly larger cohorts will have to validate the concentration ranges of metabolites reported here. Until then, this study is the most extensive set of metabolite concentrations for CSF healthy dogs reported to date. 

## Conclusions

To our knowledge, this is the first report of dogs CSF analysis through ^1^H NMR spectroscopy. In addition to that, no other study has been simultaneously quantifying and analysing correlations for as many as 13 metabolites in the same sample of dog CSF. The homogeneity of the obtained results and the low biologic variability allow for the reported data to be used as reference values in further studies and indicate that the ^1^H NMR spectroscopy analysis of dog’s CSF could help in designing and interpreting clinical and therapeutic trials in dogs with CNS pathology.
